# Paediatric germ cell tumours and congenital abnormalities: a Children's Oncology Group study

**DOI:** 10.1038/sj.bjc.6605169

**Published:** 2009-07-14

**Authors:** K J Johnson, J A Ross, J N Poynter, A M Linabery, L L Robison, X O Shu

**Affiliations:** 1 Division of Epidemiology and Clinical Research, Department of Pediatrics, University of Minnesota, Minneapolis, MN 55455, USA; 2 Masonic Cancer Center, University of Minnesota, Minneapolis, MN 55455, USA; 3 Department of Epidemiology and Cancer Control, St Jude Children's Research Hospital, Memphis, TN 37232, USA; 4 Department of Medicine, Vanderbilt Ingram-Cancer Center and Center for Health Services Research, Vanderbilt University, Nashville, TN 38105, USA

**Keywords:** germ cell tumours, paediatrics, congenital abnormalities

## Abstract

**Methods:**

Maternally reported congenital abnormalities (CAs) were examined in a case–control study of 278 cases of paediatric germ cell tumours (GCTs) and 423 controls.

**Results and conclusions:**

Germ cell tumours were significantly associated with cryptorchidism in males (OR=10.8, 95% CI: 2.1–55.1), but not with any other specific CA in either sex.

The risk factors for paediatric germ cell tumours (GCTs), which comprise a histologically heterogeneous group of tumours affecting an estimated 360 individuals <15 years of age each year in the United States (Bernstein *et al*, 1999; US Census Bureau, 2007), have not been well described (Bernstein *et al*, 1999). However, congenital abnormalities (CAs) have often been reported in association with GCTs in case series and in record linkage studies (Li and Fraumeni, 1972; Fraumeni *et al*, 1973; Li *et al*, 1973; Birch *et al*, 1982; Mann *et al*, 1993; Altmann *et al*, 1998; Little, 1999; Nishi *et al*, 2000; Merks *et al*, 2005; Bjorge *et al*, 2008; Rankin *et al*, 2008). The presence of cryptorchidism is a confirmed risk factor for testicular GCTs in men (Sarma *et al*, 2006), but its association with paediatric GCTs has not been as well studied. We evaluated the association between certain CAs and childhood GCTs by sex, age at diagnosis, anatomical location, and histology in a Children's Oncology Group (COG) study.

## Materials and methods

The study protocol was approved by the Institutional Review Boards of the University of Minnesota and by the participating COG institutions. Details regarding this study have been described previously (Chen *et al*, 2005). Briefly, GCT cases with malignant extracranial tumours, diagnosed from the time of birth to 14 years of age between 1 January 1993 and 31 December 2001, were ascertained from US and Canadian COG hospitals. Eligible diagnoses were dysgerminoma–seminoma–germinoma, embryonal carcinoma, yolk sac tumour, choriocarcinoma, malignant teratoma, and mixed GCTs. Controls were recruited through random digit dialling, and frequency was matched to cases on sex and birth year±1 year, at ratios of approximately 1 : 2 for males and 1 : 1 for females. Cases and controls were eligible if they had a telephone in their residence and if their biological mother spoke English and consented to an interview. Participation rates were 81 and 67% for cases and controls, respectively. Exposure information was collected from mothers by telephone interview, including the seven CA categories shown in Table 3.

### Statistical analyses

We used SAS version 9.1. (SAS Institute Inc., Cary, NC, USA) to conduct statistical analyses. Statistical differences in the frequency or means of sociodemographic and infant characteristics between cases and controls were assessed using Mantel–Haenszel χ^2^ tests and one-way analysis of variance. We used unconditional logistic regression to examine the associations between CAs and GCTs, adjusting for the matching factors, sex and child's age. We conducted analyses stratified by sex, age at diagnosis (⩽2, >2 years), tumour histology, and anatomical location. As the number of cases was small, the effect of potential confounding variables on risk estimates was examined by adding them one at a time to models. All statistical tests were two-sided.

## Results

In both sexes, GCTs occurred most frequently in the gonads, with yolk sac tumours being the most common histological subtype, followed by other non-seminomas in males and teratomas in females. The distribution of age at diagnosis was bimodal, with peaks observed before the age of 5 years and after the age of 10 years in both sexes (Table 1).

No statistical differences were found between cases and controls with respect to gestational age, birth weight, or maternal age. Control mothers were more educated and more likely to be of white race than were case mothers. A higher frequency of cases had household incomes below $20 000 than did controls (Table 2).

In males, there were statistically significant increased risks of GCTs in association with any CA (OR=2.5, 95% CI: 1.3–4.9), which was mainly because of cryptorchidism (OR=10.8, 95% CI: 2.1–55.1). The risk of GCTs increased significantly with increasing number of CAs in males (*P* for trend=0.01). There were no other significant associations between GCTs and CAs in either sex (Table 3). Models that included the variables shown in Table 2 did not materially change the results (data not shown).

There were no significant associations between CAs and GCTs in children ⩽2 or >2 years, except for cryptorchidism, in which risks were similar (OR_⩽2 years_=8.2, 95% CI: 0.9–72.7; OR_>2 years_=14.6, 95% CI: 1.4–152.2) (data not shown).

Individuals with any CA had a significantly increased risk for extragonadal GCTs (OR=1.7, 95% CI: 1.0–2.9), with a significantly positive linear trend with increasing number of CAs (*P*=0.009) (Supplementary Table 1). Statistically significant increased risks were observed for GCTs in association with cryptorchidism for gonadal (OR=19.9, 95% CI: 3.5–111.9) but not for extragonadal (OR=3.0, 95% CI: 0.3–34.7) GCTs. For extragonadal GCTs, significant increased risks were found in association with mental retardation (OR=15.8, 95% CI: 1.4–178.0), congenital heart defects (OR=2.7, 95% CI: 1.1–6.0), and skeletal defects (OR=5.0, 95% CI: 1.3–19.1). No significant associations were observed between GCTs and CAs in analyses stratified by histological subtype (seminoma, yolk sac tumour, teratoma, and other non-seminomas), with the exception of cryptorchidism, which increased the risk for seminomas (OR=57.0, 95% CI: 1.9–∞), yolk sac tumours (OR=11.1, 95% CI: 1.6–75.6), and other non-seminomas (OR=14.8, 95% CI: 1.5–147.4). Cryptorchidism was not reported in any males with teratomas (*n*=14) (data not shown).

## Discussion

In the largest case–control study of paediatric GCTs, we found limited evidence for an overall association between paediatric GCTs and Cas, with the exception of cryptorchidism. Other controlled studies have reported positive associations between paediatric GCTs and CAs, including any birth defect (Mann *et al*, 1993; Altmann *et al*, 1998; Bjorge *et al*, 2008), heart defects (Nishi *et al*, 2000), cryptorchidism (Nishi *et al*, 2000), and musculoskeletal/spinal abnormalities (Narod *et al*, 1997; Agha *et al*, 2005).

In males, there was an ∼11-fold increased risk of GCTs in association with cryptorchidism, which is consistent with findings from adult testicular cancer studies (Sarma *et al*, 2006). Cryptorchidism in children with GCTs has been noted previously in case series and reports (Li and Fraumeni, 1972; Huddart *et al*, 1990; Mukai *et al*, 1998) and in isolated cases in studies with comparison groups (Johnston *et al*, 1986; Wanderas *et al*, 1998). The excess risk in this study was primarily confined to gonadal tumours, with increased risks evident for seminomas, yolk-sac tumours, and other non-seminomas, which is consistent with what has been reported for adults (Coupland *et al*, 1999).

In females, there was no evidence of an association between CAs and GCTs. Reports of congenital ovarian abnormalities in association with ovarian GCTs are uncommon, with one case series study noting two cases of ovarian dysgenesis (Li *et al*, 1973); no such case was recorded in our study.

We observed an increased risk of extragonadal GCTs in individuals with reported skeletal defects, mental retardation, and congenital heart defects. Associations between skeletal and congenital heart defects and GCTs have been reported previously, but have not specified the tumour site (Narod *et al*, 1997; Nishi *et al*, 2000; Agha *et al*, 2005).

The strengths of this study include its relatively large size compared with that of previous studies and a more detailed analysis of previously reported associations. Limitations include CA ascertainment through a maternal interview that may be incomplete, especially for CAs that are minor or not clearly visible. The ascertainment of CAs may have been higher in cases because of the cancer investigations, which would produce a positive bias in the risk estimate (Rothman and Greenland, 1998). We were also limited by low statistical power to detect modest associations. Finally, selection bias may have affected our results if controls were not a representative of a sample of non-diseased individuals from the population from which cases were ascertained (Rothman and Greenland, 1998).

This study provides limited evidence for a link between most types of CAs and paediatric GCTs with the exception of cryptorchidism in males, and a possible link between certain types of CAs and extragonadal tumours.

## Figures and Tables

**Table 1:** Characteristics of paediatric GCT diagnoses

**Tumour characteristics**	**No. of males (%)**	**No. of females (%)**
*Anatomical location*		
Testis/ovary	47 (56.6)	97 (49.7)
Extragonadal	31 (37.4)	89 (45.6)
Metastatic	3 (3.6)	4 (2.1)
Unknown	2 (2.4)	5 (2.6)
		
*Histology*		
Yolk sac tumour	46 (55.4)	79 (40.5)
Teratoma	14 (16.9)	57 (29.2)
Seminoma	2 (2.4)	43 (22.1)
Other non-seminomas^a^	17 (20.5)	8 (4.1)
Other^b^	3 (3.6)	7 (3.6)
Not specified	1 (1.2)	1 (0.51)
		
*Diagnosis age category (years)*		
0–4	64 (77.1)	84 (43.1)
5–9	4 (4.8)	37 (19.0)
10–14	15 (18.1)	74 (38.0)
		
Total	83 (100.0)	195 (100.0)

GCT=germ cell tumour.

a Embryonal carcinoma, choriocarcinoma, polyembryoma.

b Mixed germ cell tumour components and malignant tumour cells.

**Table 2:** Infant and parental sociodemographic characteristics of cases and controls

**Characteristics**	**No. of cases (%)**	**No. of controls (%)**	***P*-value**
*Gestational age category (weeks)*			
<37	35 (12.6)	44 (10.4)	
37–42	236 (84.9)	367 (86.8)	
>42	7 (2.5)	12 (2.8)	0.37
Mean (s.d.)	39.5 (5.6)	39.6 (4.6)	0.76
			
*Birth weight (grams)*			
2501–4000	214 (77.0)	351 (83.0)	
⩽2500	22 (7.9)	26 (6.1)	
>4000	42 (15.1)	46 (10.9)	0.05
Mean (s.d.)	3354 (681)	3373.7 (590)	0.69
			
*Maternal age category (years)*			
⩽24	90 (32.4)	128 (30.3)	
25–29	99 (35.6)	145 (34.3)	
30–34	60 (21.6)	111 (26.2)	
⩾35	29 (10.4)	39 (9.2)	0.56
Mean (s.d.)	27.2 (5.5)	27.3 (5.4)	0.66
			
*Maternal education*			
Less than high school	28 (10.1)	23 (5.4)	
High school graduate	110 (39.7)	143 (33.9)	
College, no degree	53 (19.1)	97 (23.0)	
College degree and/or graduate school	86 (31.1)	159 (37.7)	0.004
			
*Maternal race*			
White	213 (76.9)	356 (84.4)	
Black	25 (9.0)	25 (5.9)	
Hispanic	27 (9.8)	23 (5.4)	
Other	12 (4.3)	18 (4.3)	0.05
			
*Household income (US dollars)*			
<20 000	85 (31.0)	83 (19.9)	
20 000–30 000	60 (21.9)	109 (26.1)	
30 001–50 000	63 (23.0)	124 (29.7)	
>50 000	66 (24.1)	102 (24.4)	0.03

**Table 3:** The association between paediatric GCTs and congenital abnormalities by sex

	**Males^a^**				**Females** a			
	**No. of cases (%)**	**No. of controls (%)**	**OR^b^**	**95% CI**	**No. of Cases (%)**	**No. of Controls (%)**	**OR^b^**	**95% CI**
*Any congenital abnormality*								
No	58 (71.6)	152 (84.9)	1.0	Ref.	157 (81.8)	200 (83.3)	1.0	Ref.
Yes	23 (28.4)	27 (15.1)	2.5	1.3–4.9	35 (18.2)	40 (16.7)	1.1	0.7–1.8
								
*Number of congenital abnormalities*								
0	58 (71.6)	152 (84.9)	1.0	Ref.	157 (81.8)	200 (83.3)	1.0	Ref.
1	18 (22.2)	22 (12.3)	2.5	1.2–5.2	29 (15.1)	35 (14.6)	1.0	0.6–1.8
>1	5 (6.2)	5 (2.8)	2.6	0.7–9.7	6 (3.1)	5 (2.1)	1.5	0.5–5.1
*P*-trend^c^				0.01				0.42
								
*Cryptorchidism (males)*								
No	74 (90.2)	178 (98.8)	1.0	Ref.	—	—	—	—
Yes	8 (9.8)	2 (1.1)	10.8	2.1–55.1	—	—	—	—
								
*Hernia*								
No	77 (92.8)	174 (96.1)	1.0	Ref.	191 (99.0)	232 (96.3)	1.0	Ref.
Yes	6 (7.2)	7 (3.9)	1.8	0.6–5.8	2 (1.0)	9 (3.7)	0.3	0.1–1.3
								
*Down's syndrome*								
No	83 (100.0)	180 (99.5)	—	—	191 (99.0)	240 (99.6)	1.0	Ref.
Yes	0 (0.0)	1 (0.5)	—	—	2 (1.0)	1 (0.4)	2.5	0.2–28.3
								
*Mental retardation*								
No	82 (98.8)	180 (99.5)	1.0	Ref.	193 (99.0)	241 (100.0)	—	—
Yes	1 (1.2)	1 (0.5)	2.4	0.1–41.9	2 (1.0)	0 (0.0)	—	—
								
*Congenital heart defect*								
No	78 (94.0)	174 (96.1)	1.0	Ref.	183 (93.8)	229 (95.0)	1.0	Ref.
Yes	5 (6.0)	7 (3.9)	1.5	0.5–5.1	12 (6.1)	12 (5.0)	1.2	0.5–2.8
								
*Large/multiple birthmarks*								
No	80 (96.4)	174 (96.1)	1.0	Ref.	178 (92.2)	227 (94.2)	1.0	Ref.
Yes	3 (3.6)	7 (3.9)	1.0	0.2–4.1	15 (7.8)	14 (5.8)	1.4	0.6–2.9
								
*Skeletal defect*								
No	81 (97.6)	177 (98.3)	1.0	Ref.	190 (97.4)	239 (99.2)	1.0	Ref.
Yes	2 (2.4)	3 (1.7)	1.6	0.2–10.0	5 (2.6)	2 (0.8)	3.1	0.6–16.4
								
*Other*								
No	77 (93.9)	176 (97.2)	1.0	Ref.	185 (95.8)	233 (96.7)	1.0	Ref.
Yes	5 (6.1)	5 (2.8)	3.0	0.8–11.1	8 (4.2)	8 (3.3)	1.3	0.5–3.4

CA=congenital abnormality; CI=confidence interval; GCT=germ cell tumour; OR=odds ratio.

a Data were missing on at least one CA category in males for two cases and two controls and in females for three cases and one control.

b Adjusted for child's age (except for crytorchidism in which only males were included in the analysis).

c Calculated from model that included a continuous variable for the number of birth defects.
