# Supplementary material for: Paediatric germ cell tumours and congenital abnormalities: a Children's Oncology Group study
Source: Br J Cancer. 2009 Jul 14;101(3):518–21. doi: 10.1038/sj.bjc.6605169 (PMC2720246; doi:10.1038/sj.bjc.6605169)
Supplement: Supplementary Table 1 [file 6605169x1.doc]

**Supplementary table 1.** Association between GCTs and congenital abnormalities by anatomic location.

|  |  | **Gonadal** | | | **Extragonadal** | | |
| --- | --- | --- | --- | --- | --- | --- | --- |
| **Congenital abnormality** | **No. of**  **Controls** | **No. of**  **Cases** | **OR^a^** | **95% CI** | **No. of**  **Cases** | **OR^a^** | **95% CI** |
| Any congenital abnormality |  |  |  |  |  |  |  |
| no | 352 (84.0) | 111 (78.2) | 1.0 | ref. | 92 (77.3) | 1.0 | ref. |
| yes | 67 (16.0) | 31 (21.8) | 1.4 | 0.9-2.3 | 27 (22.7) | **1.7** | **1.0-2.9** |
|  |  |  |  |  |  |  |  |
| No of congenital abnormalities |  |  |  |  |  |  |  |
| 0 | 352 (84.0) | 111 (78.2) | 1.0 | ref. | 92 (77.3) | 1.0 | ref. |
| 1 | 57 (13.6) | 26 (18.3) | 1.4 | 0.8-2.3 | 21 (17.7) | 1.5 | 0.8-2.7 |
| >1 | 10 (2.4) | 5 (3.5) | 1.6 | 0.5-4.8 | 6 (5.0)^b^ | **2.9** | **1.0-8.9** |
| p-trend^c^ |  |  |  | 0.19 |  |  | **0.009** |
|  |  |  |  |  |  |  |  |
| Cryptorchidism (males) |  |  |  |  |  |  |  |
| no | 178 (98.9) | 40 (85.1) | 1.0 | ref. | 30 (96.8) | 1.0 | ref. |
| yes | 2 (1.1) | 7 (14.9) | **19.9** | **3.5-111.9** | 1 (3.2) | 3.0 | 0.3-34.7 |
|  |  |  |  |  |  |  |  |
| Hernia |  |  |  |  |  |  |  |
| no | 407 (96.22) | 139 (96.5) | 1.0 | ref. | 116 (97.5) | 1.0 | ref. |
| yes | 16 (3.8) | 5 (3.5) | 1.0 | 0.4-2.9 | 3 (2.5) | 0.6 | 0.2-2.2 |
|  |  |  |  |  |  |  |  |
| Down syndrome |  |  |  |  |  |  |  |
| no | 421 (99.5) | 143 (99.3) | 1.0 | ref. | 118 (99.2) | 1.0 | ref. |
| yes | 2 (0.5) | 1 (0.7) | 1.4 | 0.1-15.8 | 1 (0.8) | 1.3 | 0.1-18.0 |
|  |  |  |  |  |  |  |  |
| Mental retardation |  |  |  |  |  |  |  |
| no | 422 (99.8) | 144 (100.0) | - | - | 117 (97.5) | 1.0 | ref. |
| yes | 1 (0.2) | 0 (0.0) | - | - | 3 (2.5)^d^ | **15.8** | **1.4-178.0** |
|  |  |  |  |  |  |  |  |
| Congenital Heart defect |  |  |  |  |  |  |  |
| no | 404 (95.5) | 139 (96.5) | 1.0 | ref. | 108 (90.0) | 1.0 | ref. |
| yes | 19 (4.5) | 5 (3.5) | 0.7 | 0.2-1.9 | 12 (10.0)^e^ | **2.7** | **1.1-6.0** |
|  |  |  |  |  |  |  |  |
| Large/Multiple birthmarks |  |  |  |  |  |  |  |
| no | 402 (95.0) | 132 (91.7) | 1.0 | ref. | 113 (95.0) | 1.0 | ref. |
| yes | 21 (5.0) | 12 (8.3) | 1.5 | 0.7-3.2 | 6 (5.0) | 1.2 | 0.4-3.1 |
|  |  |  |  |  |  |  |  |
| Skeletal defect |  |  |  |  |  |  |  |
| no | 417 (98.8) | 142 (98.6) | 1.0 | ref. | 115 (95.8) | 1.0 | ref. |
| yes | 5 (1.2) | 2 (1.4) | 1.2 | 0.2-6.2 | 5 (4.2)^f^ | **5.0** | **1.3-19.1** |
|  |  |  |  |  |  |  |  |
| Other |  |  |  |  |  |  |  |
| no | 410 (96.9) | 137 (96.5) | 1.0 | ref. | 112 (93.3) | 1.0 | ref. |
| yes | 13 (3.1) | 5 (3.5) | 1.06 | 0.4-3.0 | 8 (6.7) | 2.4 | 0.9-6.3 |

^a^Adjusted for child’s age and sex (except for crytorchidism where only males were included in the analysis)

^b^All cases with multiple CAs had reported congenital heart defects. Other reported defects in these cases were: Klinefelter syndrome (n=1); cleft palate/lip (n=1); skeletal defect, mental retardation and eye abnormalities (n=1); cryptorchidism (n=1); mental retardation and skeletal defect (n=1); Down syndrome, mental retardation, and strabismus (n=1)

^c^Calculated from model that included a continuous variable for the number of birth defects

^d^Tumors were located in the pelvis (n=2) and vagina (n=1)

^e^Tumors were located in the mediastinum (n=2), abdomen (n=2), pelvis (n=5), nasopharynx (n=1), vagina (n=1), and unspecified head, face or neck (n=1)

^f^Tumors were located in the pelvis (n=3), abdomen (n=1), and retroperitoneum (n=1)
